# Highly Sensitive Zinc Oxide Fiber-Optic Biosensor for the Detection of CD44 Protein

**DOI:** 10.3390/bios12111015

**Published:** 2022-11-14

**Authors:** Zhaniya U. Paltusheva, Zhannat Ashikbayeva, Daniele Tosi, Lesya V. Gritsenko

**Affiliations:** 1 Department of General Physics, Satbayev University, Satpayev Str., 22, Almaty 050013, Kazakhstan; 2 School of Engineering and Digital Sciences, Nazarbayev University, Nur-Sultan 010000, Kazakhstan; 3 Laboratory of Biosensors and Bioinstruments, National Laboratory Astana, Nur-Sultan 010000, Kazakhstan

**Keywords:** optical fiber sensors, optical ball resonators, zinc oxide, refractive index sensors, reflectometry

## Abstract

Currently, significant progress is being made in the prevention, treatment and prognosis of many types of cancer, using biological markers to assess current physiological processes in the body, including risk assessment, differential diagnosis, screening, treatment determination and monitoring of disease progression. The interaction of protein coding gene CD44 with the corresponding ligands promotes the processes of invasion and migration in metastases. The study of new and rapid methods for the quantitative determination of the CD44 protein is essential for timely diagnosis and therapy. Current methods for detecting this protein use labeled assay reagents and are time consuming. In this paper, a fiber-optic biosensor with a spherical tip coated with a thin layer of zinc oxide (ZnO) with a thickness of 100 nm, deposited using a low-cost sol–gel method, is developed to measure the CD44 protein in the range from 100 aM to 100 nM. This sensor is easy to manufacture, has a good response to the protein change with detection limit of 0.8 fM, and has high sensitivity to the changes in the refractive index (RI) of the environment. In addition, this work demonstrates the possibility of achieving sensor regeneration without damage to the functionalized surface. The sensitivity of the obtained sensor was tested in relation to the concentration of the control protein, as well as without antibodies—CD44.

## 1. Introduction

Over the past twenty years, a huge amount of innovation in optoelectronics and in the field of fiber-optic telecommunications has led to a significant reduction in the price of optical components, which has allowed fiber-optic sensors to move from the category of experimental laboratory devices to the category of widely used devices. According to forecasts, the global market for fiber-optic sensors should reach $4.9 billion by 2025 at compound average annual growth rate (CAGR) of 10.9% [1]. The alteration of one or more properties of propagating light waves (intensity, phase, polarization, or frequency) as they move through the sensing zone is the basis for the operation of fiber-optic sensors. 

Sensors using optical fiber as the sensing element most commonly use single-mode optical fibers when operating at infrared telecom wavelengths [2]. Most sensors that use single-mode fibers are intrinsic, whereas the effects of the measurand changes are transduced into an in-line optical component such as a grating [3] or a thin-film-coated plasmonic device [4]. A standard single-mode optical fiber usually consists of three layers: a core doped with materials to increase its refractive index (RI), a cladding, and a buffer coating that protects the fiber from mechanical damage [5].

Unique properties such as small size, high sensitivity, light weight, reliability and immunity to electromagnetic interference make fiber-optic sensors promising materials for applications in medicine [6], energy [7], construction [8], defense [9], and other industries [10]. In addition, the most important parameter in biomedical measurements is selectivity—the ability to detect a specific measurand of interest. Upon contact with an analyte, analysis of the transmission spectrum of an optical fiber can provide qualitative and quantitative information about the substances under investigation. To date, successful studies have been performed with respect to the detection of the biomarker of breast cancer [11], as well as the common biomarker of cancer stem cell protein coding gene CD44 [12].

This paper presents a fiber-optic biosensor for detecting the CD44 protein on a spherical tip coated with zinc oxide thin layer by the sol–gel method. Zinc oxide is of interest for biological sensing due to its many unique properties. This key technological material has become the subject of deep research in the last decade due to its unique functional and nanomorphological properties [13]. In addition, it is considered one of the leaders among oxide semiconductors due to its amazing physical and chemical properties. Semiconductor ZnO is an inorganic binary compound A^II^B^IV^ with a band gap of 3.37 eV in the near UV region of the spectrum and a high exciton binding energy of 60 meV at room temperature [14]. Such unique characteristics of ZnO have attracted considerable interest from researchers in the scientific community with the prospect of its application in various electronic and optoelectronic devices. As a wide bandgap material, ZnO shows the ability to withstand high temperatures, large electric fields, high breakdown voltages, and high-power operation. Currently, zinc oxide is actively used in semiconductor electronics as a transparent conductive metal oxide. ZnO is an n-type semiconductor due to the presence of interstitial zinc atoms and oxygen vacancies in its crystal structure. Although ZnO crystallizes in three different forms, the most thermodynamically stable under normal conditions of pressure and ambient temperature is its hexagonal wurtzite structure [15].

Zinc oxide is recognized as a promising material for applications in biosensors due to its cost effectiveness, non-toxicity, and chemical stability, as well as its high isoelectric point (IEP) of about 9.5, which makes it suitable for the adsorption of low-IEP proteins, enzymes, and DNA through electrostatic interactions [16]. Moreover, a great advantage for the development of biosensors is that ZnO has a rich morphological diversity of nanostructures [17].

Today, ZnO is widely used to detect glucose [18], cholesterol [19], uric acid [20], leptospira [21], antigens [22], ascorbic acid [23], cancer cells [24], DNA [25], and various biomarkers [26]. This semiconductor material is widely used in gas sensors [27], for targeted delivery of anticancer drugs [28], and other applications [29].

In this work, a ZnO-based fiber-optic biosensor was constructed to detect the CD44 protein using an anti-CD44 antibody. Zinc oxide thin layer was deposited on the spherical tip of the optical fiber by a low-cost, low-tech sol–gel method at room temperature, making it suitable for use in natural conditions. 

The biomarker CD44, which is expressed in many types of cancer, is an adhesion receptor that regulates the process of metastasis on the cell surface [30]. Patient CD44 studies have shown a direct correlation between serum CD44 levels and cancer occurrence in pancreatic cancer [31], lung cancer [32], breast cancer [33], prostate cancer, and head and neck carcinoma [34]. Current methods for detecting the CD44 protein are mainly based on enzyme-linked immunosorbent assay (ELISA) or other labeled methods [35]. Current CD44 detection techniques like ELISA and other immunoassay types have several drawbacks, including poor performance in terms of limit of detection (LoD) and the capacity to test just one analyte at a time. On the other hand, the method proposed in this article for detecting the CD44 biomarker with a ZnO thin layer on a spherical fiber tip features several advantages, such as reliability, speed, low cost, and label-free operation. Moreover, the optical fiber manufacturing process is one-step, which can greatly increase production volumes. Unlike existing sensors based on electrochemical interaction [36] and photoelectric interaction [37], our sensor has a lower detection limit in dilute serum from 10 aM and a wide measurement range from 10 aM to 100 nM, as well as high biocompatibility.

## 2. Materials and Methods

### 2.1. Equipment

All measurements from ball resonator optical fibers were performed using an optical backscatter reflectometer (OBR, LUNA OBR 4600) in the scan range from 1525 nm to 1610 nm with a gain of 0 dB and a resolution bandwidth of 0.258 Hz, recording 65,536 data points for each measurement. The spectra of ball resonators were demodulated using an optical backscatter analyzer (OBR; Luna OBR4600, Luna Inc., Roanoka, VA, USA). The surface morphology of a zinc oxide coated fiber-optic spherical tip was studied using a scanning electron microscope (SEM, Auriga Crossbeam 540, Carl Zeiss, Oberkochen, Germany) equipped with energy-dispersive X-ray spectroscopy (EDX, Aztec, Oxford Instruments, Abingdon, UK), which was used for elemental analysis, and also with transmission electron microscopy studies (TEM, JEOL, JEM-1400 plus, Peabody, MA, USA). To detect various characteristic functional groups, an infrared absorption spectrophotometer with Fourier transform FTIR Nicolet iS10 (Thermo Fisher Scientific Inc., Tokyo, Japan) was used. Nitrogen was used as purge gas. All measurements were carried out at room temperature 22 °C. The structural properties of the semiconductor coating were studied on a Rigaku SmartLab X-ray diffractometer (XRD, Rigaku Corp., SmartLab.,Tokyo, Japan).

### 2.2. Fabrication and Characterization of Ball Resonator Optical Fiber Sensor 

**Figure 1:**
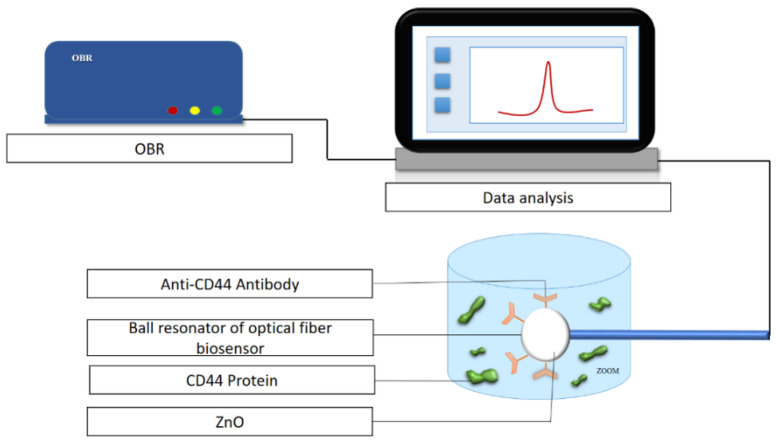
Experimental setup for the detection of the CD44 protein using a biofunctionalized sensor.

In this work, a ball resonator optical fiber sensor that worked as an ultra-weak interferometer was fabricated on a standard single-mode fiber (SMF-28) using a splicing machine (Fujikura LZM-100) that uses a CO_2_ laser as a localized heater. The splicing machine reduces the fabrication cost of the optical fiber sensor and increases its repeatability. To fabricate the ball resonator optical fiber, two SMF-28 fibers were aligned in the x-axis under the CO_2_ laser, spliced, and a high power was applied to the optical fiber to fabricate the ball structure at the tip by axis turning, and pulling in opposite directions. As a result, a spherical tip was formed at the end of the fiber, which was subjected to a break near to the splicing point [38]. The described device worked as an interferometric fiber-optic sensor. During operation, part of the optical signal propagating along the fiber is reflected at the boundary between the core and shell of the microsphere, while the rest is transmitted and is reflected from the surface of the microsphere according to the scheme presented in [38]. These two beams interfere with each other, the reflection from the surface of the microsphere depends directly on the optical properties of the deposited ZnO coating. The thus-obtained spectrum is interrogated using the OBR; this instrument is needed in order to detect low spectral fringes obtained through the weak interfering pattern in the spherical fiber tip. The experimental setup for the detection of the CD44 protein was carried out on an optical backscatter reflectometer (Figure 1). The 557–551 µm optical fiber is found for CD44 protein detection, while the 514–519 µm and 525–521 µm optical fibers are used for prostate-specific antigen (PSA) protein measurement control and CD44 protein negative control measurement, respectively. The values of absolute and relative power, rotation speed and feeding speed to obtain the spherical tip sensors of diameters 557–551 µm, 525–521 µm, 514–519 µm are shown in Table S1 [39].

**Figure 2:**
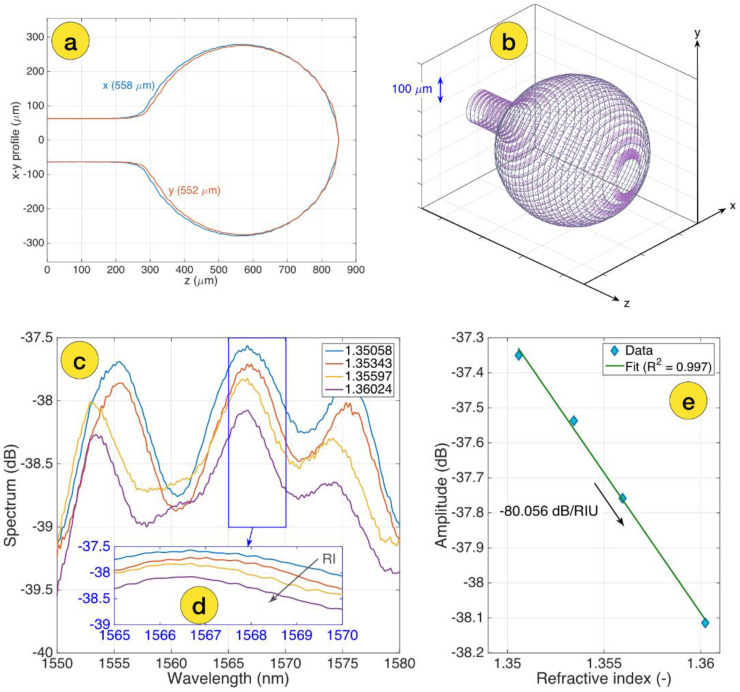
Design and calibration of the ball resonator biosensor: (**a**) two-sided profilometry of the ball resonator, measured through the CO_2_ laser splicer; (**b**) 3D mesh of the ball resonator, highlighting the longitudinal cross-sections (grey) and azimuthal planes (purple); (**c**) S-polarization spectrum of the bare ball resonator sensor, measured for different RI values ranging from 1.351 to 1.360; (**d**) the inset shows the spectral feature around 1567 nm used for spectral tracking of the intensity; (**e**) RI calibration, showing the change of intensity as a function of the RI change and sensitivity estimation (−80.056 dB/RIU).

Before and after zinc oxide thin layer coating, the sensors were calibrated in air, in distilled water, and in several concentrations of the sucrose solutions by measuring the spectrum change according to the refractive index (RI) change. The initial concentration of sucrose solution was 10%, and was increased stepwise by adding 400 µL of 40% sucrose solution up to 5 concentration points, with RI values from 1.351 to 1.360 [40]. Figure 2a shows two-sided profilometry of the spherical-tipped ball resonator used to detect the CD44 protein in this work and a 3D mesh of the ball resonator with longitudinal cross-sections and azimuthal planes (shown in Figure 2b).

Polarization P and S spectra were selected for the data analysis. Spectral patterns were identified using a feature tracking method that highlights the most significant spectral responses. Figure 2c shows the S-polarization spectrum of a bare-ball resonator probe measured for various RI values ranging from 1.351 to 1.360, as well as the cumulative spectral response in the range where the probe has the greatest response for sensitivity estimation, as shown in Figure 2d and amplitude change as a function of RI change. The curve processed with linear regression, R^2^ = 0.997 with an estimated sensitivity −80.056 dB/RIU (shown in Figure 2e).

### 2.3. Ball Resonator Optical Fiber Surface Coating with the ZnO Thin Layer

Before coating with zinc oxide, the surface of the ball resonator optical fibers was cleaned in 20 mL of Piranha solution (H_2_SO_4_:H_2_O_2_ = 4:1) for 20 min to remove organic impurities and increase -OH groups. Then, the sensors were washed with distilled water and dried with nitrogen gas, followed by silanization in a 5% solution of (3-aminopropyl) trimethoxysilane (APTMS) in methanol solution for 30 min. Next, the optical fibers were heat treated for 60 min at 100 °C and then coated with a layer of zinc oxide using the sol–gel method [41]. The sol was deposited by dissolving 0.4 g of zinc acetate Zn(CH_3_COO)_2_ (purity 98%, Sigma-Aldrich, St. Louis, MO, USA) in 10 mL of ethanol at room temperature with vigorous stirring on a magnetic stirrer for 1 h. Afterwards, the sol solution was uniformly applied to surface of the ball resonator by dip-coating technique and dried in a muffle furnace at 100 °C for 10 min. Subsequent annealing at 250 °C for 60 min resulted in the formation of a uniform ZnO thin layer on the surface of the spherical tip. The ZnO immobilization on the surface of the ball resonator optical fiber was verified by scanning electron microscopy (SEM), and transmission electron microscopy (TEM).

### 2.4. Functionalization of the ZnO-Based Ball Resonator Optical Biosensor with CD44 Antibody

To immobilize the CD44 antibody on the surface of the ball resonator optical fiber, the carboxyl groups were introduced to the ZnO thin layer by immersing the sensors in the 11-mercaptoundecanoic acid solution for 16 h and activated by the solution of 1-ethyl-3-(3-dimethylaminopropyl)carbodiimide hydrochloride (250 mM)/ N-hydroxysuccinimide (100 mM) (EDC/NHS) for 15 min. Finally, 4 µg/mL of CD44 antibody was immobilized onto the ball resonator optical fiber’s surface for 1 h and the unreacted biomolecules were blocked with the 10% poly(ethylene glycol) methyl ether amine solution. The same functionalization protocol was followed for the control prostate-specific antigen (PSA) and negative control which only excluded the step of antibody immobilization and was blocked after the EDC/NHS activation. All sensors were stored at 2–4 °C. 

### 2.5. CD44 Detection

The fabricated ball resonator optical fiber biosensors were employed for the detection of CD44 protein diluted in human serum. For this, the transducer part of the optical fiber biosensor decorated with zinc oxide thin layer and CD44 antibody bioreceptor was immersed in the analyte solution with CD44 protein, while the other end of the optical fiber was connected to the OBR instrument. The concentration range of CD44 analyte was from 100 aM up to 100 nM at 10× dilution. Spectra of ball resonators were demodulated using an optical backscatter reflectometer (OBR). The OBR made it possible to register the spectral response during analyte–bioreceptor bindings over 10 min with every minute recordings. The CD44 detection performance were validated on three sensors.

### 2.6. Specificity Analysis

The most crucial feature of the biosensors is the specificity which is defined as an ability of a bioreceptor to differentiate between target and control analytes. The specificity of the optical fiber biosensor was justified by the PSA detection at the same concentration ranges as of CD44 protein from 100 aM to 100 nM. Moreover, the measurements of negative control in the absence of antibody were conducted by ball resonator optical fiber biosensor. The sensitivity parameters were assessed comparing the response to CD44 to the responses obtained with the controls.

### 2.7. Regeneration Studies

To investigate the reusability of the ZnO-based optical fiber biosensor, the sensor was regenerated employing 10 mM Glycine-HCl solution with pH of 3. The biosensor after the CD44 protein detection was placed into the Glycine-HCl solution for 5 min and washed with PBS. Afterwards, the regenerated sensor was used again for the CD44 analyte measurements. The ZnO-based optical fiber biosensor was regenerated three times to validate the sensor’s performances.

## 3. Results and Discussion

### 3.1. Surface Characterization of Ball Resonator Optical Fiber Biosensor

**Figure 3:**
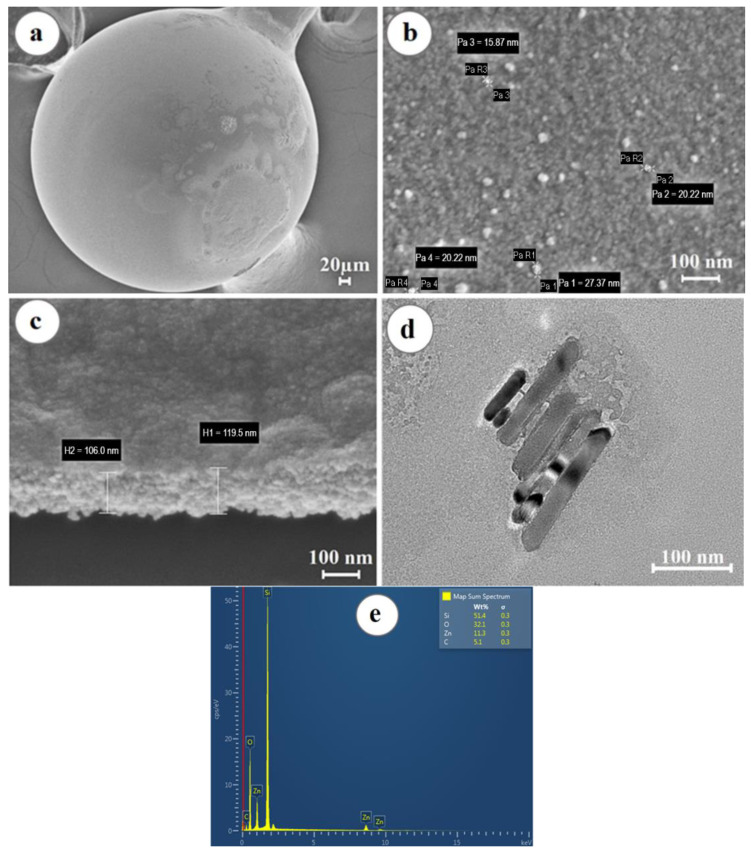
Morphological analysis of the surface of the ball resonator optical fiber coated with a ZnO layer: (**a**) SEM image of the optical fiber sensor showing its spherical shape and surface coating; (**b**) SEM image demonstrating the grain structure of the ZnO layer; (**c**) ZnO layer thickness on the surface of the optical fiber biosensor; (**d**) TEM image of ZnO; (**e**) EDS—surface analysis of the ball resonator optical fibers demonstrating the presence of Zn, O, C, and Si elements.

The results of the study of electron microscopy showed that the microsphere has a regular spherical shape, while zinc oxide forms a uniform layer that evenly covers the surface of the spherical resonator (shown in Figure 3a), consisting of grains with a diameter of 10 ± 5 nm, as shown in Figure 3b. 

The thickness of the deposited ZnO thin layer was about 100 nm (shown in Figure 3c). Transmission electron microscopy studies confirmed the SEM results, as shown in Figure 3d. The elemental composition of the samples was studied by the method of energy dispersive X-ray spectroscopy (shown in Figure 3e). The EDS X-ray fluorescence spectrum showed the presence in the sample of Zn and O atoms in the amount of 11.3% and 32.1%, respectively, forming the ZnO layer, as well as Si and C atoms, representing the elemental basis of the resonator.

### 3.2. Characterization of Synthesized ZnO Thin Layer

**Figure 4:**
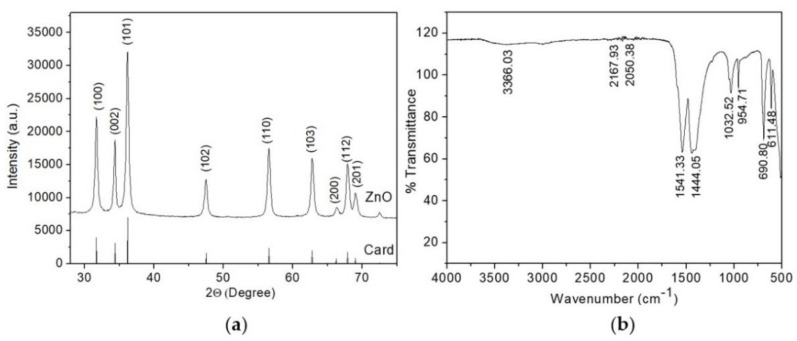
(**a**) X-ray diffraction pattern of ZnO layer; (**b**) FT-IR spectrum of ZnO layer.

The results of the structural properties of the semiconductor coating studied on an X-ray diffractometer are shown in Figure 4a. X-ray diffraction analysis confirmed the formation of crystalline zinc oxide. All diffraction peaks at (2θ) angles of 31.73°, 34.4°, 36.21°, 47.49°, 56.52°, 62.8°, and 67.87° correspond to reflection from (100) crystal planes, (002), (101), (102), (110), (103), and (112), respectively, the hexagonal structure of zinc oxide wurtzite (space group P63mc [42]). It is noted that good crystallinity was achieved, since all diffraction reflections coincide with the reference sample, whose lattice parameters are a = 3.2539 Å, b = 3.2539 Å, c = 5.2098 Å (JCPDS card no. 01-080-0075).

According to the Debye–Scherrer equation [43,44], the sizes of zinc oxide crystallites were estimated based on XRD analysis data:

D = kλ / β cos θ(1)

where k = 0.89 is the Scherrer constant (dimensionless coefficient), θ is the diffraction angle, β is the peak width at half maximum expressed in radians, λ = 1.54 Å is the CuKα radiation wavelength. Calculations performed for the most intense peak (101) showed that the average grain size of ZnO layer along this direction was about 12 nm, which is in agreement with the electron microscopy data.

The synthesized zinc oxide thin layer was subjected to FT-IR analysis to detect various characteristic functional groups. The FT-IR spectrum of ZnO thin layer in the wavenumber range from 500 to 4000 cm^−1^ is shown in Figure 4b. The peaks indicate the characteristics of the functional group present in the synthesized zinc oxide thin layer. It was found that the samples have absorption peaks in the range of 3366.03 cm^−1^, 2167.93 cm^−1^, 2050.38 cm^−1^, 1541.33 cm^−1^, 1444.05 cm^−1^, 1032.52 cm^−1^, 954.71 cm^−1^, 690.80 cm^−1^, 611.48 cm^−1^. The broad peak at 3366.03 cm^−1^ corresponds to the O-H stretching vibration of the amide group. The peaks at 2167.93 cm^−1^ and 2050.38 cm^−1^ represent the H-O-H vibrations of the crystallization water molecule cluster. The peaks at 1541.33 cm^−1^, 1444.05 cm^−1^, 1032.52 cm^−1^, 954.71 cm^−1^ correspond to C=C alkane group stretch, C=C aromatic ring stretch, and polyphenol (C=O), C–H bending vibration of the alkane group, C–N stretching, and C–H bending vibration, respectively [45,46]. The absorption peaks at 690.80 cm^−1^, 611.48 cm^−1^ correspond to the metal-oxygen vibration mode (stretching vibrations of ZnO).

### 3.3. Detection of CD44 Biomarker

**Figure 5:**
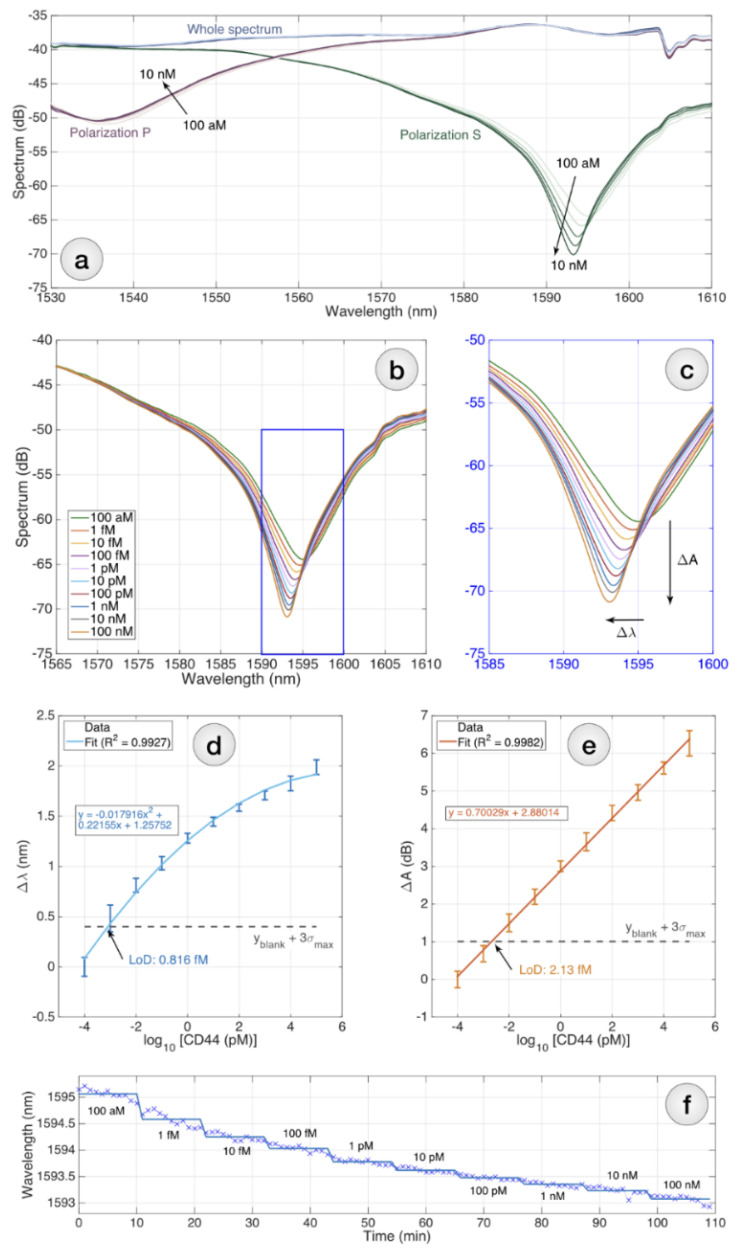
Detection of CD44 using the biofunctionalized sensor: (**a**) reflective spectrum of the biosensor; (**b**) the S-polarization spectrum is chosen for interrogation; (**c**) the inset shows the spectral portion in correspondence to the main dip; interrogation can be performed by tracking either the intensity change ΔA or the wavelength shift Δλ; (**d**,**e**) CD44 protein detection charts, reporting the wavelength shift (**d**) and the intensity change (**e**); (**f**) sensorgram for the CD44 detection, reporting the instantaneous wavelength (markers) and average (solid lines) over 109 min with 1-min sampling.

There are two basic processes that can be applied for the optical fibers’ surface modification: physical adsorption and covalent attachment. In this study, covalent attachment was performed using an intermediate layer of linkers that react with both zinc oxide and the bioreceptor. The resulting functionalized biosensors were used to detect the CD44 protein (sensor 557–551 µm), control measurement of PSA protein (sensor 514–519 µm) and negative control (sensor 525–521 µm). An OBR was used to detect the signal when measuring the CD44 protein in the concentration range from 100 aM to 100 nM (Figure 5). 

At the same time, the functionalized ball resonator was immersed in a vial containing 250 µL of a CD44 protein solution. Measurements for each concentration were carried out for 10 min. A total of 12 measurements, including all concentrations of CD44 protein, human serum, and PBS solution, were performed at each concentration. Figure 5a–c show the spectral response sampled after 5 min of S(green) and P (purple)polarization, for RI changes registered for CD44 concentration from 100 aM (bright curves) to 10 nM (dark curves), as well as wavelength shift diagrams (Figure 5d) and intensity change, measured for each CD44 concentration ranging from 100 aM to 100 nM (error bars = ±standard deviation reported over 11 consecutive measurements sampled every 1 min). The spectral response in the range where the sensor had the most sensitive response was integrated to determine the LoD of the sensor, which was calculated to be 2.13 fM (Figure 5e). The LoD was estimated using the method proposed by Chiavaioli et al. [47]: LoD = f ^−1^ ($y_{\mathrm{blank}}$ + $3\sigma_{\max})$; where $y_{\mathrm{blank}}\;$is the blank response hereby recorded at the lowest concentration of CD44, when the sensor is barely responsive, and $\sigma_{\max}$ is the maximum standard deviation.

The CD44 protein for each concentration and for control measurements was prepared with the addition of serum. The spectral envelope corresponding to the incidence point was fitted to a second-order polynomial equation by quadratic regression using the equation:$\;y=a_{2}x^{2\;}+a_{1}x+a_{0}$, where x is the log10 CD44 concentration in nM and y is the spectral response of the sensor in unit s db. The dip wavelength has been estimated by finding the minimum of y, i.e., $-a_{1}/\left(2a_{2}\right)$; the minimum spectral level is the amplitude in correspondence of the minimum, i.e., $\left(a_{0}-a_{1}{}^{2}\right)/\left(4a_{2}\right)$.

### 3.4. Specificity Analysis

For specificity analysis, PSA protein measurement in the same concentration range as for CD44 protein was used as a control. Functionalization of the sensor was carried out in the same order as for the main protein CD44 sensor. Unlike the primary sensor, where there is an increasing response, the control sensor with PSA protein does not cause a significant signal change, and concentration-dependent trend was not seen. 

**Figure 6:**
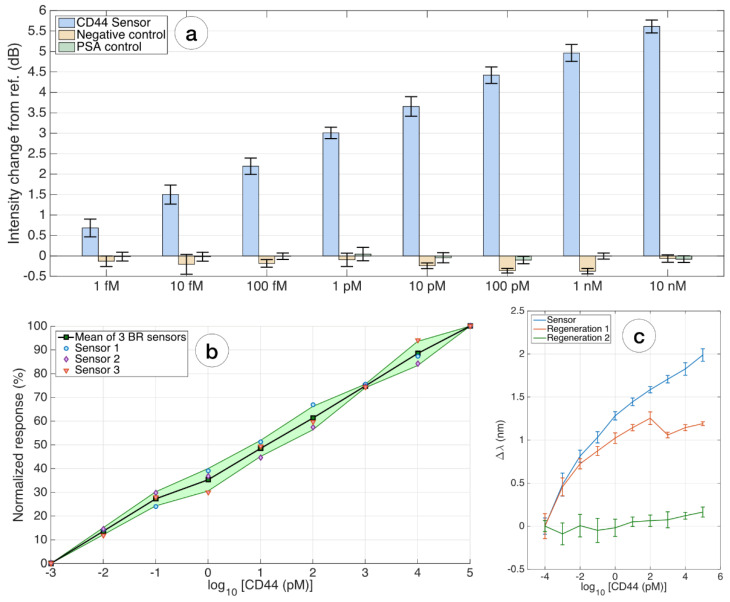
Evaluation of specificity, repeatability, and regeneration of the biosensor: (**a**) the specificity was estimated by comparing the response of the CD44 biosensor from the reference value, for every concentration within 1 fM and 10 nM, to two additional ball resonator sensors fabricated with the same method and having a similar sensitivity and interrogation process. Negative control = biosensor with no antibodies; PSA control = CD44-biosensor detecting different contrations of PSA. Error bars = ±standard deviation reported over 11 consecutive measurements; (**b**) repeatability of the whole biosensing device; the chart compares the normalized response, in terms of intensity change, for 3 different ball resonator biosensors fabricated and interrogated with the same method, responding to CD44 concentrations. Furthermore, 0–100% normalized values account for the response of the sensor at 1 fM (≈LoD) and 100 nM, respectively. Solid line = average, shadowed region = ±standard deviation for 3 different sensors with same biofunctionalization; markers show individual data points; (**c**) regeneration of the biosensor: the chart displays the response of the same biosensor in the first measurement, compared with the sensor regenerated once (red) and twice (green).

Moreover, sensor specificity was assessed by comparing the response of the CD44 biosensor to the reference value for each concentration of the biosensor without CD44 antibodies (negative control). The amplitude values of the sensor without antibodies range from negative to positive values and do not have a distinct change in amplitude associated with an increase in protein concentration (Figure 6a).

The repeatability of the entire biosensor design is demonstrated on three biosensors fabricated and functionalized by the same method and presented as a diagram. Figure 6b shows normalized sensor responses from 0–100% at 1 fM (≈LoD) and 100 nM, respectively, with markers indicating individual data points, mean, and standard deviation for the 3 sensors. All sensors showed the same trend at the different sensitivities.

### 3.5. Regeneration

In this work, the regeneration of the interaction between the main analyte (CD44 protein) and the ligand was carried out, allowing the functionalized sensor surface to be used twice for measurements. To do this, the biosensor was immersed in a 10 mM Glycine-HCl regeneration buffer for 5 min on a magnetic stirrer. A low pH of 3.0 was used, because, at low pH, proteins become partially unfolded and positively charged. 

The binding sites of proteins repel each other, and the unfolding promotes further separation of the molecules. Figure 6c shows the response of the same biosensor after the first and second regenerations. It is noted that this method of regeneration shows good repeatability for the first regeneration; after the secondary regeneration, the response of the biosensor is practically absent.

## 4. Conclusions

This paper presents the first biosensor using a fiber-optical ball resonator with a uniform zinc oxide thin layer deposited by the sol–gel method as a sensor platform for detecting the CD44 protein. The advantages of this biosensor include the ease of manufacture on a standard and cheap telecommunication fibers in one step, and the simplicity of applying a ZnO thin layer by the sol–gel method without the use of expensive equipment. Moreover, this biosensor provides a good response, demonstrating an increase in spectral amplitude with increasing analyte concentration in diluted serum, enabling the detection of concentrations as low as few femtomolars; high sensitivity to changes in the refractive index of the environment, making it suitable as a biosensor platform after functionalization; excellent reproducibility demonstrated on three sensors with different sensitivities; and the possibility of sensor regeneration without damaging the functionalized surface. The resulting biosensor showed almost zero response to the control PSA protein and to the biosensor without antibodies—CD44. These biosensor characteristics represent a promising new way of detecting the important CD44 biomarker in cancer diagnostics.

## Data Availability

Not applicable.
